# Immune response associated with ischemia and reperfusion injury during organ transplantation

**DOI:** 10.1007/s00011-022-01651-6

**Published:** 2022-10-25

**Authors:** Qiao Tang, Chong Dong, Qian Sun

**Affiliations:** 1 Department of Anesthesiology, Renmin Hospital of Wuhan University, No.238 Jiefang Road, Wuchang District, Wuhan, 430060 China; 2 Organ Transplantation Center, Tianjin First Central Hospital, Tianjin, 300192 China; 3 Tianjin Key Laboratory for Organ Transplantation, Tianjin, 300192 China

**Keywords:** Ischemia and reperfusion injury, Organ transplantation, Innate immunity, Adaptive immunity

## Abstract

**Background:**

Ischemia and reperfusion injury (IRI) is an ineluctable immune-related pathophysiological process during organ transplantation, which not only causes a shortage of donor organs, but also has long-term and short-term negative consequences on patients. Severe IRI-induced cell death leads to the release of endogenous substances, which bind specifically to receptors on immune cells to initiate an immune response. Although innate and adaptive immunity have been discovered to play essential roles in IRI in the context of organ transplantation, the pathway and precise involvement of the immune response at various stages has not yet to be elucidated.

**Methods:**

We combined “IRI” and “organ transplantation” with keywords, respectively such as immune cells, danger signal molecules, macrophages, neutrophils, natural killer cells, complement cascade, T cells or B cells in PubMed and the Web of Science to search for relevant literatures.

**Conclusion:**

Comprehension of the immune mechanisms involved in organ transplantation is promising for the treatment of IRI, this review summarizes the similarities and differences in both innate and adaptive immunity and advancements in the immune response associated with IRI during diverse organ transplantation.

## Introduction

Organ transplantation has emerged as the most direct and effective clinical option for patients suffering from terminal organ failure and there is an expanding worldwide demand for organ transplants. Actually, cell damage caused by organ resection and storage can unquestionably deteriorate transplanted organs and may be a contributing factor for clinical outcomes and prognosis [1]. The period between the cessation of blood and the proceed of organ cold perfusion is widely recognized as warm ischemia time [2, 3]. Organs from donation of cardiac arrest death (DCD) are vulnerable to warm ischemia for a longer duration than those from donation of brain death (DBD) because hypoperfusion and warm ischemia begin quite a long time before cardiac arrest, when circulatory and respiratory function gradually fails after drug withdrawal [3]. Cold ischemia time is the period between cold perfusion and blood supply regeneration following transplantation [4]. Prolonged cold ischemia impacts transplanted organs’ functional recovery and long-term survival [5]. Reperfusion injury aggravates tissue injury by restoring blood perfusion and oxygen supply to transplanted organs. We have learned that ischemia and reperfusion injury (IRI) can spark apoptosis of tubular cells, resulting in severe renal function damage, which is the primary driver of delayed graft function (DGF) or even chronic graft injury [6]. Furthermore, every effort should be made to avoid mechanical damage and destruction of donor organs during the procurement process.

**Fig. 1:**
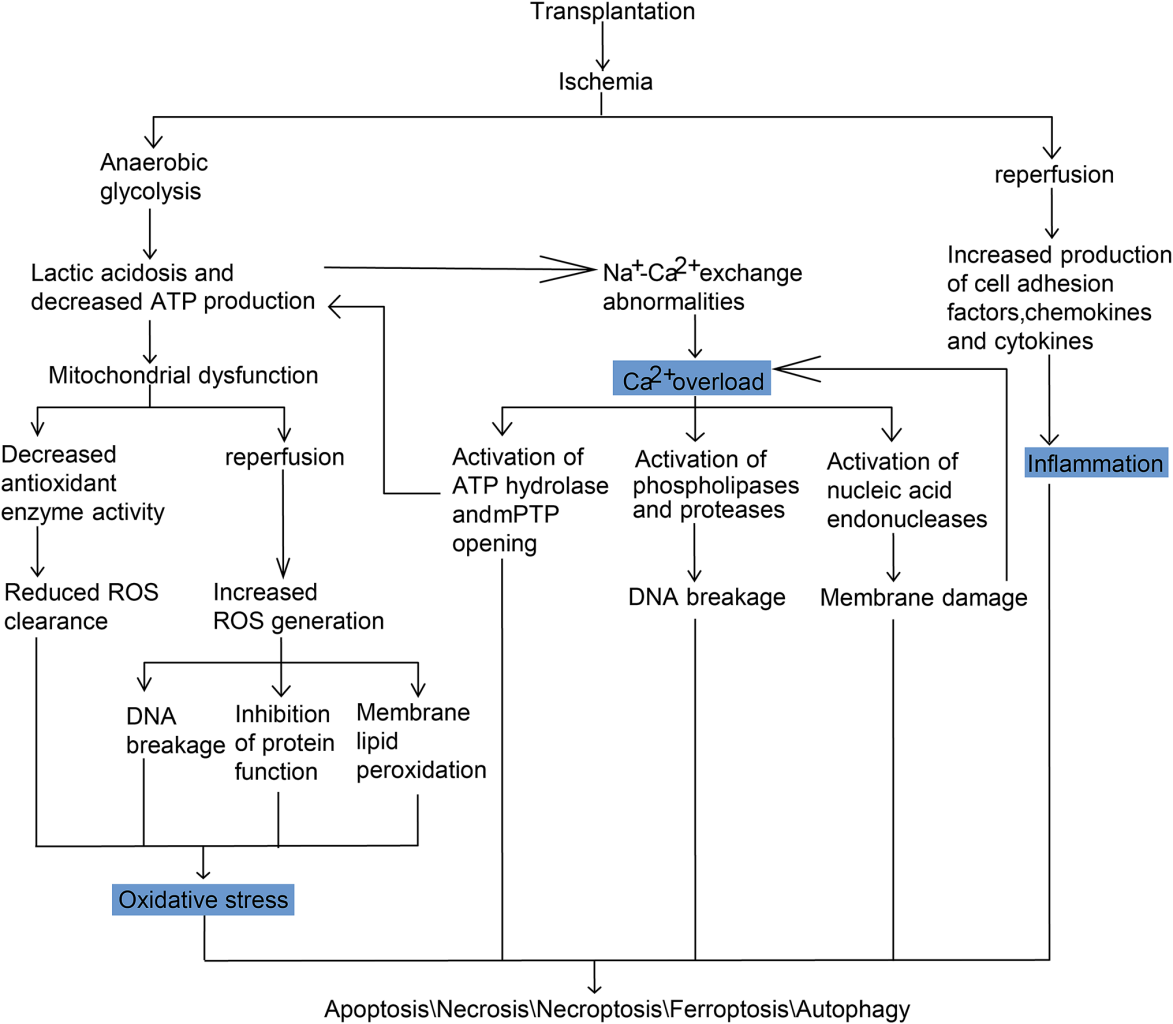
Mechanisms of ischemia and reperfusion-mediated cellular injury in the context of organ transplantation. Oxidative stress, calcium overload and excessive inflammatory reaction play the important roles in the pathogenesis of IRI. First, hypoxia and ischemia during organ donation accelerate the conversion of transplanted cells to anaerobic metabolism, which results in lactic acidosis, decreases adenosine triphosphate (ATP) production, and causes mitochondrial dysfunction. Second, excessive Ca^2+^ and reactive oxygen species (ROS), along with extracellular and intracellular signaling molecules, are crucial in IRI-mediated cell destruction in the case of organ transplantation. Finally, severe IRI in the context of organ transplantation causes different forms of cell death, which make a significant contribution to immune activation

IRI is an inherent immune-related pathophysiological process that occurs in the process of organ transplantation. Ischemia causes microvascular function impairment and metabolic disorders like oxygen abnormality as well as pH abnormality in transplanted organs, and succeeding reperfusion causes calcium disorder and an increase in oxygen free radicals (Fig. 1), facilitating immune response and cell death pathways [7]. Over the last few decades, several immune activation pathways have been unearthed, with some cell types specifically performing opposing pro-inflammatory and anti-inflammatory capabilities in an IRI-dependent pattern. The identification of novel molecular events and the immune regulatory mechanisms associated with IRI establishes a link between immune response and organ regeneration, allowing for long-term transplant function [8]. Despite the clinical characteristics of different organ transplantation appear distinct, IRI-related immune response is mediated by similar mechanisms in common. This paper reviews the effects and progress of IRI mediated by various factors in different organ transplantation in terms of the mechanism of IRI triggering immune response, innate immunity and adaptive immunity.

## Innate immunity

### Danger signals for initiating innate immune response associated with IRI during organ transplantation

IRI-induced cell death stimulates the release of cellular components such as heat shock protein (HSP) as well as high mobility group box (HMGB) in transplanted organs, as supported by the research below, and these components serve as damage-associate molecular patterns (DAMPs) [9, 10]. Gene expression of HSP27 and 90 induced by IRI is up-regulated after pancreatic transplantation, providing a very promising prospect for improving pancreatic IRI after transplantation [11]. In canine pancreas autotransplantation, compared to the straightforward University of Wisconsin (UW) preservation approach, two-layer preservation method reduces IRI due to the high level of HSP60 expression [12]. According to a recent study, HMGB-1 levels are elevated in patients of IRI in the context of liver transplantation [13]. For a rat liver transplantation model, pretreatment of DCD-derived grafts with soluble thrombomodulin improves IRI by reducing HMGB-1 and inflammatory factors namely tumor necrosis factor (TNF)-α and interleukin (IL)-6 [14]. A growing stack of research links mitochondrial DNA (mtDNA) with DAMPs since the worsening of IRI is accompanied with the increase of mtDNA [15]. In the experimental model, senile donor animals treated with lytic drugs which could clear away the senescent cells, reduce the release of mtDNA and aseptic inflammation, thereby extending the life expectancy of senile cardiac allografts, as compared with young donor animals [16]. Overall, animal research findings corroborate the apparent participation of DAMPs in the initiating innate immune response associated with IRI during organ transplantation, but its relevance remains disputed in the lack of clinical trials.

**Fig. 2:**
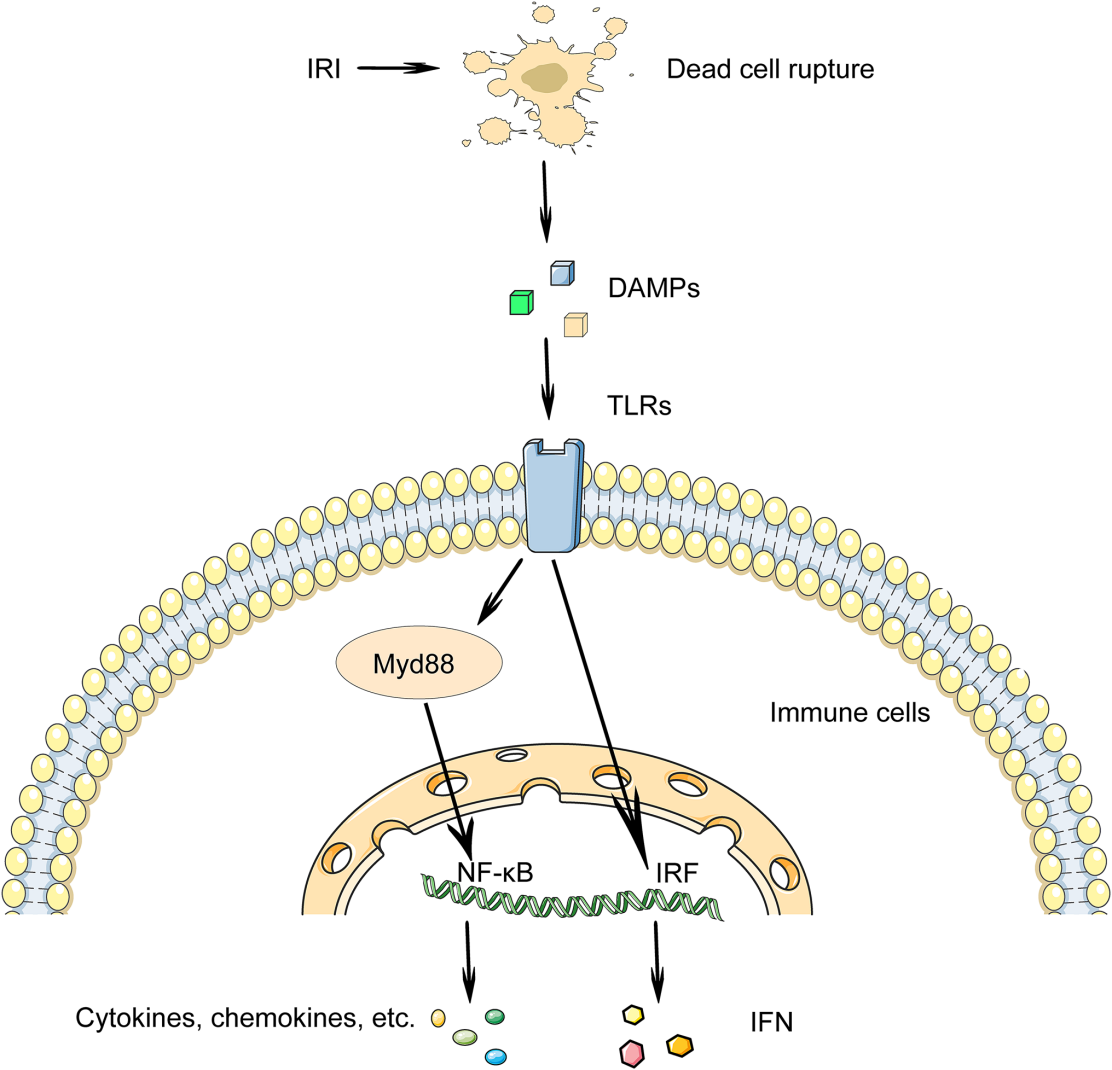
Specific recognition and binding of Toll-like receptors (TLRs) by damage-associate molecular patterns (DAMPs) triggers the initiation of the immune system. Ischemia and reperfusion-mediated cell rupture releases endogenous DAMPs. In the immune cells, TLRs are activated by DAMPs, which stimulates signaling pathways involving interferon regulatory factor (IRF) and marrow differentiation factor 88 (Myd88)/NF-κB signaling pathways, causing the release of cytokines and chemokines such as interferon (IFN) and interleukin (IL)

Toll-like receptors (TLRs) displayed in immune cells react to DAMPs, and their activation stimulates transcription factors involving interferon regulatory factor (IRF) and nuclear factor-κB (NF-κB) signalling pathways (Fig. 2), causing the release of interferon (IFN)-α/β and IL-1, as well as the production of IRI-induced aseptic inflammation [17]. Thirteen TLRs have been found in mammals, with TLR3, 7, 8, and 9 residing intracellularly, while TLR1-6 and TLR10 are reported on the cell membrane surface [18]. Importantly, some TLRs (mainly TLR2 and TLR4) clearly influence the pathological development of IRI, and they are triggered by DAMPs generated during ischemia, initiating innate immune response [19]. Inhibition of TLR2 by pretreatment with TLR2 monoclonal antibody possesses notable survival benefit against IRI and reduces TLR2-mediated cytokine production in a mouse kidney transplantation model [20]. TLR4 activation promotes the release of pro-inflammatory mediators, the migration and infiltration of leukocytes, the activation of the innate and adaptive immune systems, the maintenance of tubular necrosis, and the enhancement of renal fibrosis during IRI of the transplanted kidney [21]. A clinical experiment shows that TLR4, which binds to HMGB-1, is significantly raised due to ischemic injury and the functional deletion of TLR4 mutation is related to less pro-inflammatory gene expression, which offers compelling evidence for demonstrating the pathogenesis of IRI during human kidney transplantation related to the expression of TLR4 in donor kidney cells [22]. Cold IRI of transplanted kidney can also be improved by reducing NF-κB phosphorylation to inhibit the TLR4/marrow differentiation factor 88 (Myd88) pathway and reducing downstream inflammation like TNF-α, IL-1, and IL-6 [23]. Furthermore, IRI activates TLR3 following RNA release in the transplanted heart, and TLR3 deletion protects the heart from IRI [24]. As a consequence, according to the recent data, targeted TLR3 therapy, in addition to TLR2 and 4, has emerged as a novel treatment proposal for halting IRI in organ transplant recipients. But the transformation and application of TLRs therapy in IRI following organ donation is still one issue that has to be addressed, and further evidence is needed.

### Macrophages

**Fig. 3:**
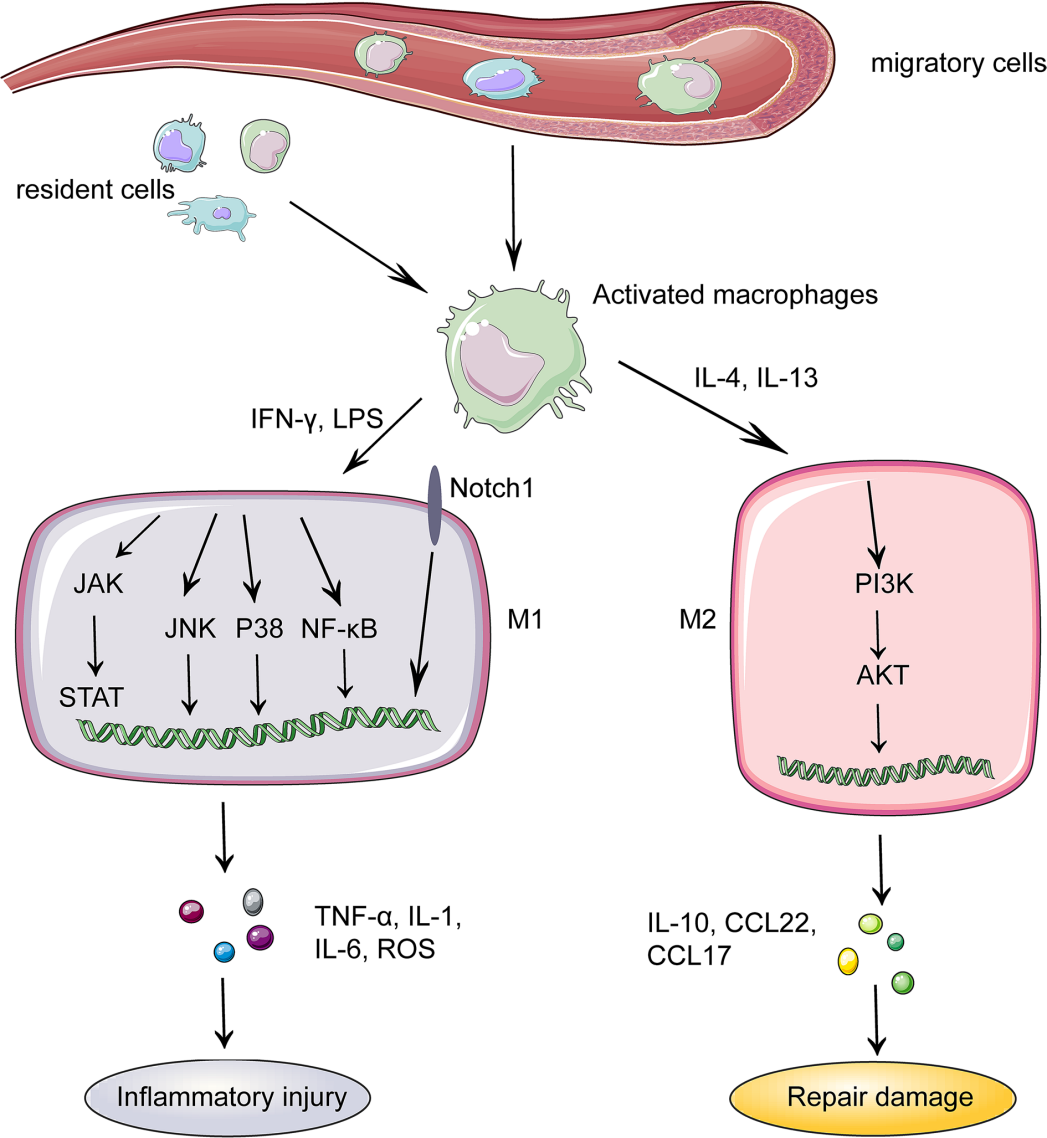
Polarization of macrophages and its molecular mechanism. The migratory macrophages and resident macrophages are activated and differentiated into macrophages with different functions in IRI region under the influence of different molecules. Interferon (IFN)-γ or lipopolysaccharide (LPS) can polarize macrophages into M1 which produces inflammatory factors such as tumor necrosis factor (TNF)-α, interleukin (IL)-1, IL-6 and reactive oxygen species (ROS) through c-Jun N-terminal kinase (JNK), Notch, Janus kinase signal transducers and activators of transcription (JAK/STAT), and NF-κB signaling pathways. IL-4 or IL-13 activates M2 mainly through phosphoinositide 3-kinase (PI3K)/protein kinase B (AKT) pathway, and M2 generates immune molecules such as IL-10, chemoattractant cytokine ligand (CCL)22 and CCL17 to repair the damage

Emerging evidence suggests that macrophages participate in the pathophysiology of IRI during organ transplantation. Macrophages are made up of both resident and migratory cells. During thoracic organ transplantation, resident immune cells in IRI are activated, promoting the creation of a pro-inflammatory milieu, and subsequently immune cells from the circulation are recruited to augment and maintain the immunological cascade [25]. Numerous experimental evidence in renal transplantation also illustrates that macrophages influx following renal reperfusion may contribute to IRI-mediated acute kidney injury by secreting cytokines, attracting neutrophils, and triggering apoptosis [26]. Furthermore, Kupffer cells (KCs), also known as hepatic resident macrophages, can take the lead in sensing early extracellular DAMPs and be activated to yield chemokines and cytokines including such IL-1, 6, 8, 12, and TNF-α, eventually resulting in IRI-mediated aseptic inflammation of transplanted liver [27]. In the context of transplantation, activated macrophages are polarized into two subsets, which are M1 mediated the initiation and maintenance of inflammation and M2 performed in inflammation regression [28]. M1 macrophages, also known as classically activated macrophages, are formed as a result of the interaction of IFN-γ/ lipopolysaccharide (LPS) and TLRs (Fig. 3). Ischemia-mediated up-regulation of HMGB1 in combination with TLR4 activates macrophages in organ transplantation, thus releasing inflammatory factors and excessive reactive oxygen species (ROS) to promote organ damage [29]. They typically have elevated levels of CD86, inducible nitric oxide synthase, as well as inflammatory components like TNF-α, IL-1 and IL-6, all of which aggravate IRI and graft injury [30]. Besides, selective inhibition of histone deacetylase (HDAC) significantly reduces cell death and improves organ function after IRI, possibly because HDAC3 is recruited to activate transcription factor 2 (ATF2) binding sites in the process of LPS activating macrophages, which activates inflammatory gene expression [31–33]. Notably, the deacetylase activity of HDAC3 interacts with the nuclear receptors coactivator 1 and 2 to specifically bind ATF3, which inhibits polarization toward M1 [34]. Exposure to IL-4 or IL-13 results in the formation of M2 macrophages, also widely recognized as alternately activated macrophages. Contrary to M1 induction of Th1 immune response, M2 macrophages prompt Th2 immune response, which is accompanied by high representation of CD163 and 206, arginase-1, inflammatory domain molecule 1, chitinase 3 protein 1, and other markers [35]. Furthermore, M2 can secrete anti-inflammatory substances like IL-10, as well as chemokines such as chemoattractant cytokine ligand (CCL)22 and CCL17, which have the function of repairing damage [36]. IRI significantly increases the likelihood of graft malfunction, graft rejection and organ failure, therefore the involvement of macrophages in all parameters connected to IRI should be deeply investigated.

The regulatory framework network of macrophage polarization encompasses c-Jun N-terminal kinase (JNK), phosphoinositide 3-kinase (PI3K), protein kinase B (Akt), Notch, Janus kinase signal transducers and activators of transcription (JAK/STAT), and NF-κB signaling pathways [37]. In the case of IRI after treatment, the expressions of JNK, p38, and NF-κB-related proteins in macrophages are sharply down-regulated, along with the lessen secretion of M1 phenotype, which is emphasizing the significance of M1 polarization in immune function [38]. Besides, liver IRI addressed with PI3K inhibition reflects obvious damage manifestations such as edema and cytoplasmic vacuolation. Activation of the PI3K-AKT pathway can reduce apoptosis and inflammation of transplanted organs, which may be attributed to the promotion of M2 polarization of macrophages [39]. Inhibiting the Notch1 as well as NF-κB pathways can decrease M1 polarization and inflammation, while increasing M2 phenotypic markers [40]. Notch1 signaling pathway is regulated in IRI-stressed orthotopic liver transplantation (OLT) treated with serelaxin via mitigating macrophage infiltration and activation, constraining pro-inflammatory cytokine, and optimizing IRI [41]. As the same, the expression of JAK2 and STAT3 is weakened in OLT treated with propofol, and IRI-mediated inflammation, oxidative stress, and apoptosis are all diminished in the hippocampus [42], inferring that IRI can activate the JAK2/STAT3 signaling pathway and boost M1 polarization during liver transplantation. In the rat model of OLT established after suberoylanilide hydroxamic acid treatment, OLT-induced IRI is alleviated by impairing the AKT/GSK3β/NF-κB pathway and reducing the M1 polarization [43]. In curcumin-pretreated liver transplantation model, the remodeling of the polarization of KCs contributes to the improvement of IRI and overall survival, which may be related to NF-κB suppression as well as peroxisome proliferator-activated receptor γ (PPARγ) initiation [44]. Ischemia induces an acidic microenvironment because of anaerobic glycolysis as well as lactic acid accumulation, which promotes M1 polarization and inhibits M2 polarization in macrophages by regulating PPARγ [45]. Indeed, understanding and identifying the mechanisms of macrophage polarization hold great promise for developing immunotherapy strategies on IRI during organ transplantation.

### Neutrophils

Neutrophils belong to immune regulatory network that allow the patients to modulate IRI during organ transplantation. A multi-step process is comprised of recruitment, migration, activation and release of granzyme, in which neutrophils gather around the region of IRI and amplify tissue damage [46]. Firstly, leukocytes in the region of IRI discharge inflammatory mediators; Secondly, vascular endothelial cells upregulate adhesion molecules; Finally neutrophils rely on integrin transfer out of the circulatory system [47]. Many studies have shown that inhibiting selectin can lower neutrophil infiltration and inflammatory response, which can benefit IRI during transplantation [48–50]. A clinical observation shows that genes producing adhesion molecules and integrins are up-regulated following cold IRI in the human liver graft [51] and integrin blocking can prevent the development of lung IRI, which is an efficient therapeutic protocol for primary graft dysfunction (PGD) of transplantation [52]. PGD is one of the leading causes of severe complications and increased mortality in transplant recipients. In a rat kidney transplantation model after cold and warm ischemia, intercellular adhesion molecule-1 (ICAM-1) inhibition reduces neutrophil infiltration, resulting in less graft damage and DGF [53]. Blocking vascular cell adhesion molecule-1 (VCAM-1) in vitro limits neutrophil recruitment and migration, reducing the degree of renal injury in IRI model [54]. Platelet-endothelial cell adhesion molecule-1 (PECAM-1) expression can increase blood neutrophils to the peak at 2 h after reperfusion, while the morphological changes in the ultrastructure of IRI-mediated cell damage are the most observable [55]. Above all, inhibiting neutrophil recruitment provides a foundation for improving IRI during organ transplantation. Chemokines are the primary components that entice neutrophils to migrate to inflammatory lesions induced by IRI. IRI rapidly stimulates the production of neutrophil chemokines like macrophage inflammatory protein-2, as well as other mediators such as C5a, to boost migration to transplanted organs [56, 57]. A study using a model of heart transplantation-mediated IRI have discovered that CXC chemokine (CXCL) 2 and 5 can modulate neutrophil extravasation and migration to inflammatory regions in cardiac grafts [58]. Furthermore, recent research indicates that the recruitment and migration of neutrophils during IRI of organ transplantation can be mediated by the TLRs signaling pathway [58, 59]. IRI promotes neutrophil activation by increasing their size, forming neutrophil clusters, and producing more elongated neutrophils [60]. The critical involvement of neutrophils in graft IRI has been acknowledged in numerous investigations, including the above-mentioned literature, while the underlying mechanism of neutrophil recruitment during IRI remains unknown. As a result, we address multiple routes of neutrophil recruitment into donated organs, which may provide new therapy options for IRI-mediated immune damage during organ transplantation.

The precise mechanism of neutrophil-mediated tissue damage in graft IRI requires further investigation. However, previous evidence indicates that increasing pro-inflammatory cytokines, ROS production, and proteases (such as elastase, cathepsin G, and myeloperoxidase) are the recognized approaches for neutrophils to promote organ IRI [61–64]. A new mechanism, neutrophil extracellular traps (NETs), has been unearthed in recent years. The accumulation of NETs in animal orthotopic lung transplantation model after long-term cold ischemia and in lung transplant patients has been confirmed, indicating that NETs are a promising therapeutic target [65]. Another study discovers that the production of NETs during PGD after lung transplantation are triggered via the attachment of TLR9 signal pathway to mtDNA released during lung IRI [66]. Experiments on rat liver transplantation reveal that preventing the formation of NETs reduces liver IRI [67]. Furthermore, perioperative DNAse administration, which depletes NETs, improves graft function following IRI, which is linked to the initiation of adaptive immune response [68]. To build a therapeutic framework for reducing IRI during organ transplantation, more and more explorations on immune responses and injury mechanisms of neutrophils are emerged.

### Natural killer cells and natural killer T cells

Natural killer (NK) cells, as effector cells of innate immune response, can exert cytotoxic effects without being energized by antigens, and they can excrete inflammatory molecules and directly destroy cells to empower with immune supervision and regulation. Animal studies reveal that TNF-related apoptosis-inducing ligand expression defends damage by controlling NK cell cytotoxicity and differentiation, as well as enhancing IFN-γ release in the IRI model [69]. Infiltration of NK cells around blood vessels and interstitium of transplanted kidney increases significantly after long-time cold ischemia [70]. Removing NK cells from graft significantly ameliorates liver IRI by reducing neutrophil infiltration and proinflammatory mediators [71]. It has been reported that NK cells infiltrate the kidney after IRI, mediating tubular epithelial damage through NK group 2 member D (NKG2D)/retinoic acid early induced transcript-1 pathway, and at the same time the reduction of NK cells can inhibit renal IRI [72]. Additionally, NK cells cause chronic damage of transplanted kidney, which avoids acute organ rejection due to T cell tolerance [73]. The above evidence confirms that NK cells, as essential immune cells, are closely linked to emergence and progression of IRI, but the mechanisms involved in the pathological process of IRI need to be clarified further in the setting of organ transplantation.

Natural killer T (NKT) cells are a special T cell subpopulation with both T cell and NK cell receptors, which can identify the lipid antigen delivered by CD1d to arouse cytotoxic activity and a diverse range of immune responses [74, 75]. Antibody-targeted deletion of NKT cells reveals a decrease in serum alanine aminotransferase, as the same in warm IRI, which is encouraging immunotherapy in the clinical outlook for transplantation [76]. NKT cells are roughly divided into two subsets: type I NKT (iNKT) cells and type II NKT (dNKT) cells. In the mouse liver IRI model, sulfatide-mediated activation of dNKT cells results in iNKT cells inactivation and improves liver IRI by inhibiting IFN-γ secretion [77]. It has been reported that iNKT cells rely on NADPH oxidase and IL-17 production to facilitate lung dysfunction and inflammation following IRI [78]. Finally, as we learn more about the immune mechanism of NKT cells in IRI, we will be able to use NKT cell-induced immune tolerance as a new therapeutic strategy for preventing transplant rejection.

### The complement cascades

The complement system regulates the IRI-mediated DGF, the loss of transplanted organs, and the risk of rejection, so a better understanding of the complement system’s activation and immune function can really help expand the blueprint for organ transplantation therapy [79, 80]. During transplantation, overactivation of donor and recipient complement system gives rise to graft injury, with graft ischemia and subsequent reperfusion being the most important mechanisms that trigger complement activation [81]. The pathways of complement activation vary with organ type during transplantation, and mainly include classical pathway, alternative pathway and lectin pathway [82]. Lung function in lung transplant recipients decreases immediately following reperfusion and is improved rapidly upon administration of C1-esterase-inhibitor [83]. In mouse heart transplantation model, inhibition of all or alternative complement pathways diminishes early complement accumulation in the graft, results in a substantial reduction in myocardial IRI by lowering the amount of inherent immune cell infiltration as well as inflammatory cytokine and adhesion molecule gene expression [84]. Factor B deficiency in the recipient's alternative pathway protects the transplanted kidney from IRI and inflammation [85]. Mannose-binding lectin (MBL) staining of human pretransplant and posttransplant kidney biopsies shows that MBL pathway in ischemia-damaged kidney is initiated at the early stage of IRI [86]. Besides, DBD increases complement activation when compared to living donor [87]. DBD aggravates myocardial IRI after transplantation in mice and human, and reduces the survival rate of mouse allografts [88]. The aforementioned clinical and experimental studies support complement activation associated with IRI during transplantation, but more research on the immune effect of complement cascade in grafts is becoming crucially influential because of the scarcity of related complement therapy drugs in the clinics.

Important complement activation products, including C3a, C5a, and membrane attack complex (MAC), exert immune effects, and regulate inflammation via conjugating to receptors to initiate immune cells and behaving as facilitators of downstream immune mechanisms [89, 90]. The C3a and C5a signaling pathways effect organ transplantation-mediated IRI through enhancing the formation of pro-inflammatory cytokines, the initiation and invasion of innate immune cells, and the commencement of adaptive immunity [91]. Nebulizing C3a receptor antagonist before transplantation significantly reduces lung IRI from DBD, supporting the role of complement inhibition in improving post-transplant IRI in the context of DBD [92]. The results of a study demonstrate that pre-ischemic treatment with C5a receptor antagonists significantly reduces tissue IRI and do not impair the formation of MAC [93, 94]. Interestingly, studies have found that C3a and C5a levels do not change significantly during the reperfusion process, and the MAC is primarily associated with post-transplant graft function [95]. After kidney transplantation from DBD and DCD, soluble C5b-9 (sC5b-9) shows marked intravenous release immediately following reperfusion but no sC5b-9 or C5a is released from the living donor kidney [96, 97]. MAC itself directly induces cell injury and necrosis in the graft by forming a transmembrane channel through a hydrophilic junction with the cell membrane [98, 99]. In general, the specific effect of each complement molecule on IRI in the case of organ transplantation has not been fully clarified.

## Adaptive immunity

### T cells-mediated adaptive immunity

In addition to the innate immune response, emerging evidence indicates that T cells serve an antigen-independent function in IRI during organ transplantation (Table 1), though the pathways by which antigen-specific T cells are operated in aseptic IRI inflammation is unknown. In one experiment involving rat liver transplantation, sotraustaurin-treated rats of liver transplantation show longer survival times and decrease T cell counts [100]. CD4^+^ T cells rapidly permeate the transplanted lung after reperfusion, but it appears that the donor factor has a greater effect on the extent of injury at the early stage of reperfusion, whereas T cells in the recipient chiefly foster the injury at the late stage of reperfusion, which may be mediated by the official launch of IFN-γ by activated T cells [101]. By combined use of gene therapy, blocking drug, and gene targeting mice, the findings demonstrate that blocking the CD154-CD40 signal prevents T cells infiltration and thus improves hepatic IRI [102]. T cells-mediated adaptive immunity has a significant impact on IRI during various organ transplantation, and the related immune mechanism warrants further investigation.

**Table 1:** Summary of references for evidence of T-cell role in IRI models

Organ	Model	Related findings	Reference
Liver transplantation for 30-h cold ischemia	Rats T spleen cells	Inhibition of T-cell activation reduces hepatocyte damage	[100]
Lung transplantation of cold ischemia for 12 h and reperfusion for 2 or 12 h	Rats	Recipient CD4^+^ T cells infiltrate lung grafts within 1 h of reperfusion and upregulate CD25 expression for the following 12 h; The role of T cells is independent of neutrophil recruitment and activation	[101]
Liver of partial 90-min warm hepatic ischemia followed by 6 h of reperfusion	Mice	CD154-CD40 T-cell signaling is the mechanism of IRI and disruption of CD154 signaling alleviates liver injury	[102]
Kidney for 32-min ischemia and 24-h reperfusion	Mice	Lack of T and B cells, no protection by A2A agonists; Associated with IFN-γ	[105]
Liver transplantation	Patients	Protective magnesium treatment is associated with reduced Th1-derived cytokines and elevated Th2-derived cytokines	[106]
Liver transplantation for 20-h ischemia	Mice	Th17 differentiation is suppressed in the ameliorated IRI	[107]
Hearts for 8-h cold ischemia before transplantation	Mice	Blocking NKG2D in γδ T cells improves cellular performance	[108]
Kidney transplantation	Patients	Infiltration of regulatory T cells into dead donor kidneys; Co-culture with regulatory T cells reduces renal cell injury	[109]
Kidney transplantation for 7-h cold ischemia	Rats	rATG reduces CD4^+^, CD8^+^ T cell infiltration and IRI-mediated apoptosis	[110]
Liver transplantation for 24-h cold ischemia	Mice	Increased tissue damage and CD8^+^ T cells in B7-H1 KO grafts	[111]
Heart transplantation for 8-h cold ischemia	Mice	Elevated levels of IL-17A produce mainly by γδ T cells after IRI	[112]
Kidney for 45 min of ischemia and 24/72 h of reperfusion	Mice	Renal impairment after IRI exacerbated by T-cell clearance with anti-CD25	[113]
Kidney for 45-min ischemia	Mice	Targeted lymphocyte therapy alters the repair of IRI	[114]
Kidney for 30-min warm ischemia	Mice	TIM-1 is expressed on activated CD4^+^ T cells after ischemic injury; Anti-TIM-1 antibody has no effect on IRI in RAG^−/−^ animals lacking T cells; Anti-TIM-1 antibody reduces CD4^+^ T cell infiltration in ischemic kidneys and improved IRI	[115]
Liver transplantation for 20-h cold ischemia or 90-min warm ischemia	Mice	Disruption or blockade of CD4^+^ T cell-dependent TIM-1 signaling pathway reduces IRI-mediated apoptosis or necrosis	[116]
Liver transplantation for 90-min ischemia and 6 h of reperfusion	Mice	IPC attenuates IRI injury associated with TIM-1 inhibition	[117]
Liver transplantation for 20-h cold ischemia	Mice	Increased TIM3 expression in CD4^+^ T cells infiltrating IR;. Targeting TIM3 accelerates IRI	[118]

T cells contributing to the occurrence and progression of IRI encompass not only CD4^+^ T cells, but also CD8^+^ T and γδ T cells, all of which have different effects in different organs and phases of the disease [103]. First, activated CD4^+^ T cells differentiate into three major subtypes of different immune functions and phenotypes: Th1 that secretes IFN-γ, Th2 that secretes IL-4 or IL-13, and Th17 that secretes IL-17, according to the cytokines they produce and damage the graft through cytokine-mediated inflammation [104]. One experiment shows that in IFN-γ knockout mice, renal IRI is more severe than that in the control group [105]. It has been demonstrated that magnesium pretreatment improves reperfusion syndrome and enhance Th2 cell activity, with increased IL-4 and IL-10, causing a shift in Th1-Th2 cytokine balance to Th2 in patients undergoing liver transplantation [106]. MicroRNA-155 deficiency reduces IRI in mice after liver transplantation, which is related to the reduction of IL-17 secretion caused by inhibition of Th17 differentiation [107]. It has been confirmed that NKG2D blockade significantly recovers damage in heart transplant models with IRI, and its effect is related to the reduction of T cell infiltration that produces IL-17 [108]. According to one study, the presence of forkhead box P3 (FoxP3) Th2 cells, as well as upregulation of Th17-related retinoid-related orphan receptor-γt mRNA in the donor kidney after cold ischemia, regulate the immune injury [109]. Previous research has already proven that various cell types differentiated by CD4^+^ T cells play distinct roles, and that balancing CD4^+^T cells differentiation also contributes to IRI-mediated graft outcome. Next, CD8^+^ T cells are also involved in IRI-mediated immune response during organ transplantation. In post-treatment ischemic transplanted kidney mice, the quantity of transplanted infiltrated CD8^+^ T cells is effectively diminished, lessening IRI-induced graft abnormality [110]. Using B7 homolog 1 (B7-H1) knockout mice, the experimental data shows that the loss of B7-H1 in liver transplantation significantly aggravates cold IRI, which is linked to a higher frequency and an absolute number of CD8^+^ T cells in both the donor and recipient [111]. Finally, IL-17A, mainly produced by γδT cells, is increased following IRI during transplantation. Neutralizing antibodies against IL-17A inhibit cardiomyocyte caspase-3 activity-related apoptosis to improve IRI after myocardial transplantation [112]. It has been proved that regulatory T cells contribute to IRI of renal transplantation [113]. During recovery of IRI after renal transplantation, regulatory T cells can be regulated to minimize fibrosis, ameliorate tubular injury and enhance growth factor yield [114].

Furthermore, T cell immunoglobulin domain and mucin domain (TIM) family is a protein encoded by T cells comprised of eight members, three of which are reported to be found: TIM1, 3, 4. Ischemic injury induces the production of TIM-1 in activated CD4^+^ T cells, and blocking TIM-1, which hinders ischemic necrosis, leukocyte enrollment and the output of regional pro-inflammatory cytokines after reperfusion, substantially prolongs survival after IRI [115]. In the background of liver transplantation, TIM-1 antibody therapy limits Tbet transcription, elevates caspase-3 activity, enhances the selectivity of Bcl-2/Bcl-xl expression, and eliminates considerable IR-induced hepatocyte necrosis/apoptosis [116, 117]. TIM-3 is displayed by stimulated CD4^+^ T cells entering the cold IRI-stressed OLT, and interrupting the TIM-3 signal polarizes the phenotype towards Th1/Th17, inhibits Th2-related FoxP3, and magnifies hepatocyte IRI, confirming its immunomodulatory character in IRI of liver transplants [118]. TIM pathway in IRI necessitates more clinical and experimental evidence, as it may be a therapeutic target for improving graft outcomes.

### B cells-mediated adaptive immunity

Although little research has paid insights into the issue of B cells in IRI, B cells gradually prove to be a component of the immune mechanism of IRI [119]. Experimental data show that compared with wild-type mice, B-cells deficient mice have functional protection for IRI [120]. When compared to the non-ischemic intestine, the B-cell chemokine CXCL13 is discovered to be ubiquitously expressed in the IRI region, as well as the B-cell-specific CXC chemokine receptor 5 (CXCR5) transcript, indicating that the intestinal tract affected by IRI expresses the chemokine CXCL13 and that attracting CXCR5 B cells into the inflammatory region is conducive to antibody-independent damage [121]. A recent study provides evidence for rapid up-regulation of the chemokine CXCL13 after renal IRI which is confirmed to be connected with the timeframe of graft ischemia in the mouse model [122]. Furthermore, the repair capacity of B cells in IRI and organ transplantation appears to be contradictory in the literature. B cells travel to inflamed tissues during the repair stage of post-ischemic organs, which can increase atrophy and aggravate functional impairment by reducing tubular proliferation, demonstrating their new position in the repair of warm IRI [123]. According to transcriptome analysis of kidney transplant biopsies, B cells contribute to the pathogenesis of advanced immune-mediated graft fibrosis and are closely related to long-term clinical outcomes of the graft [124]. Therefore, the immune effect and oriented mechanism of B cells in IRI should be given more consideration when it comes to organ transplantation.

## Conclusions

Organ transplant research over the last few decades is highly concentrated on post-transplant patients’ management especially immunosuppression. IRI is a major contributor to transplanted organ dysfunction and rejection. Although innate and adaptive immunity have been discovered to play essential roles in IRI during organ transplantation, the pathway and precise involvement of the immune response at various stages has not yet to be elucidated. Finally, we cannot deny that post-transplant rejection is the predominant cause of long-term graft survival and chronic nonfunctioning. Perhaps the perioperative IRI-induced early graft immune response seems to have a role in transplant rejection. An in-depth examination of the involvement of immune system, as well as a better knowledge of the interaction of various immune cells, would aid in clarifying the complicated immunity mechanism of IRI during organ transplantation. Moreover, it is also significant in the formation of new diagnostic and therapeutic procedures, as well as in the prevention and treatment of early peri-operative graft rejection.
